# Disruption of Membrane Integrity as a Molecular Initiating Event Determines the Toxicity of Polyhexamethylene Guanidine Phosphate Depending on the Routes of Exposure

**DOI:** 10.3390/ijms23063289

**Published:** 2022-03-18

**Authors:** Jeongah Song, Kyung-Jin Jung, Mi-Jin Yang, Woojin Kim, Byoung-Seok Lee, Seong-Kyu Choe, Seong-Jin Kim, Jeong-Ho Hwang

**Affiliations:** 1 Animal Model Research Group, Korea Institute of Toxicology, Jeongeup 56212, Korea; jeongho.hwang@kitox.re.kr; 2 Bioanalytical and Immunoanalytical Research Group, Korea Institute of Toxicology, Daejeon 34114, Korea; jungk@kitox.re.kr; 3 Jeonbuk Pathology Research Group, Korea Institute of Toxicology, Jeongeup 56212, Korea; mjyang@kitox.re.kr; 4 Toxicologic Pathology Research Group, Korea Institute of Toxicology, Daejeon 34114, Korea; woojinkim@kitox.re.kr (W.K.); bslee@kitox.re.kr (B.-S.L.); 5 Department of Microbiology, Wonkwang University School of Medicine, Iksan 54538, Korea; seongkyu642@wku.ac.kr; 6 Department of Biomedical Science, Graduate School, Wonkwang University, Iksan 54538, Korea; mbunker1.21@gmail.com

**Keywords:** polyhexamethylene guanidine phosphate, membrane integrity, route of exposure, lung injury, liver toxicity

## Abstract

Polyhexamethylene guanidine phosphate (PHMG-P), a cationic biocide, is widely used in household products due to its strong bactericidal activity and low toxicity. However, it causes fatal lung damage when inhaled. In this study, we investigated why PHMG-P causes fatal lung injury when inhaled, and demonstrated that the disruption of membrane integrity through ionic interaction—a molecular initiating event of PHMG-P—determines toxicity. Mice were injected intravenously with 0.9 or 7.2 mg/kg PHMG-P (IV group), or instilled intratracheally with 0.9 mg/kg PHMG-P (ITI group); they were euthanatized at 4 h and on days 1 and 7 after treatment. Increased total BAL cell count and proinflammatory cytokine production, along with fibrotic changes in the lungs, were detected in the ITI group only. Levels of hepatic enzymes and hepatic serum amyloid A mRNA expression were markedly upregulated in the 7.2 mg/kg IV and ITI groups at 4 h or day 1 after treatment, but returned to baseline. No pathological findings were detected in the heart, liver, or kidneys. To simulate the IV injection, A549, THP-1, and HepG2 cells were treated with PHMG-P in cell culture media supplemented with different serum concentrations. Increased serum concentration was associated with an increase in cell viability. These results support the idea that direct contact between PHMG-P and cell membranes is necessary for PHMG-induced toxicity.

## 1. Introduction

A tragic humidifier-disinfectant-associated incident occurred in Korea in 2011. Humidifier disinfectants were commonly used to prevent the growth of microorganisms in humidifier tanks in Korea. Lee et al. [1] and Kim et al. [2] reported that humidifier disinfectants can be inhaled in the form of aerosol particles smaller than 100 nm when the humidifier is in operation. Polyhexamethylene guanidine phosphate (PHMG-P), one of the main ingredients of the widely used humidifier disinfectants, was reported to cause persistent pulmonary inflammation and fibrotic changes when inhaled [3,4]. After the outbreak of this fatal lung disease, 1033 people, including fetuses, died, and more than 3285 people suffered from lung injuries, including lung disease, asthma, bronchiectasis, pneumonia, bronchitis, upper respiratory tract disease, and toxic hepatitis, as of 31 October 2021 [5,6,7,8,9]. However, some victims have complained of cardiovascular, kidney, musculoskeletal, and skin disease, but these diseases have not been recognized as humidifier-disinfectant-related diseases due to the lack of direct medical relationship between humidifier disinfectant and these diseases. The Ministry of Environment is still collecting epidemiological and toxicological studies and clinical results in order to expand the knowledge of diseases related to humidifier disinfectants other than lung diseases.

PHMG-P is a member of the polymeric guanidine family. Guanidine is a strong organic base that binds to the anionic groups—including phosphates, carboxylates, and metals—of many biomolecules [10]. Notably, owing to their unique features, synthetic molecules and drugs bearing guanidine moieties have been the subject of intense research interest. Guanidine forms non-covalent interactions with proteins and other molecular targets, leading to their inactivation and changes in biological properties [10]. The cationic nature of the guanidine family disrupts bacterial cell membranes through ionic interactions, perforates the membrane, and causes the leakage of the intracellular contents; this is the antimicrobial mechanism of the guanidine family biocides [11,12]. Our previous study proved that the positive charge of PHMG-P caused cytotoxicity in eukaryotic cells and zebrafish through the same mechanism, i.e., disturbing the plasma cell membranes’ integrity via electrostatic interactions [13].

Route of exposure is one of the most important factors that influence chemical toxicity. The most common routes by which toxicants enter the body are oral, intravenous (IV), intramuscular (IM), intraperitoneal (IP), subcutaneous (SC), and inhalation. The rate of absorption among the routes of entry generally occurs in the following order (from fastest to slowest): IV > inhalation > IM > IP > SC > oral route [14,15]. Therefore, toxicity is likely to be highest in the IV and inhalation exposure and lowest in the oral exposure. In the IV exposure, the toxic effects are rapid, and there is great potential for multiple organ exposure; when injected intravenously, toxicants directly course through the systemic circulation and are distributed to all tissues [15]. Absorption in the lungs is efficient and fast because they have a large surface area and high blood flow [14]; when inhaled, toxicants enter the upper respiratory tract or the lungs, are absorbed into the blood, and are then circulated and distributed to organs that have an affinity for them. On the other hand, once toxicants enter the gut wall through the oral or IP routes, they may be biotransformed by the liver and then circulated throughout the body.

PHMG has been widely used as a biocide in household products due to its low toxicity and strong bactericidal activity [16,17]. However, it causes fatal lung damage when inhaled. We wondered why PHMG-P causes much higher pulmonary toxicity than oral toxicity. In this study, we compared the toxicity of PHMG-P depending on the routes of exposure, via intratracheal instillation (ITI) and IV injection, and investigated whether PHMG-P causes respiratory damage when given by other routes than inhalation, and whether PHMG-P induces damages to organs other than the lungs. In addition, we demonstrated that the disruption of membrane integrity—a molecular initiating event of PHMG-P—determines toxicity according to the route of exposure.

## 2. Results

### 2.1. Body and Organ Weights

To investigate the influence of the exposure route on the PHMG-P toxicity, body weights were measured. The mice given 7.2 mg/kg PHMG-P IV (7.2 mg/kg IV) and 0.9 mg/kg PHMG-P ITI (0.9 mg/kg ITI) rapidly lost weight from day 1 to day 7, whereas no weight loss was observed in the mice given 0.9 mg/kg via the IV route (0.9 mg/kg IV) (Table 1). Although the mice in the 0.9 mg/kg ITI group received eight times less test material than those in 7.2 mg/kg IV, they lost approximately twice as much weight (11.3% in 7.2 mg/kg IV and 24.9% weight loss in 0.9 mg/kg ITI compared to the control, respectively) by day 4.

**Figure 1:**
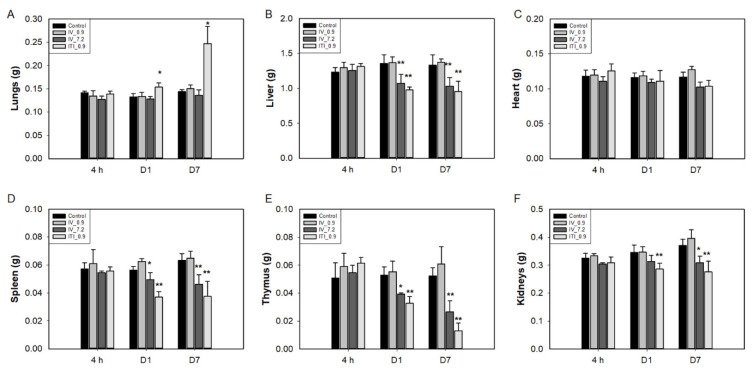
Changes in the absolute organ weights of mice: Mice were intravenously injected with 0.9 mg/kg or 7.2 mg/kg of PHMG-P or intratracheally instilled with 0.9 mg/kg of PHMG-P. Mice were sacrificed at 4 h and days 1 and 7 after treatment. Lungs (**A**), liver (**B**), heart (**C**), spleen (**D**), thymus (**E**), and kidneys (**F**). Bars represent the mean ± standard deviation (*n* = 9). Values are significantly different from the control group: * *p* < 0.05, ** *p* < 0.01.

The weights of the liver, spleen, and thymus were significantly decreased in the 7.2 mg/kg IV and 0.9 mg/kg ITI groups on days 1 and 7 (Figure 1). Lung weight increased only in the 0.9 mg/kg ITI group on days 1 and 7. Relative liver weight (organ weight/body weight) decreased in the 7.2 mg/kg IV and 0.9 mg/kg ITI groups on day 1, and in the 7.2 mg/kg IV group on day 7 (Supplementary Figure S1). Kidney weight decreased in 7.2 mg/kg IV and 0.9 mg/kg ITI groups on days 1 and 7, but relative kidney weight was not different between groups.

### 2.2. Histopathological Evaluation

**Figure 2:**
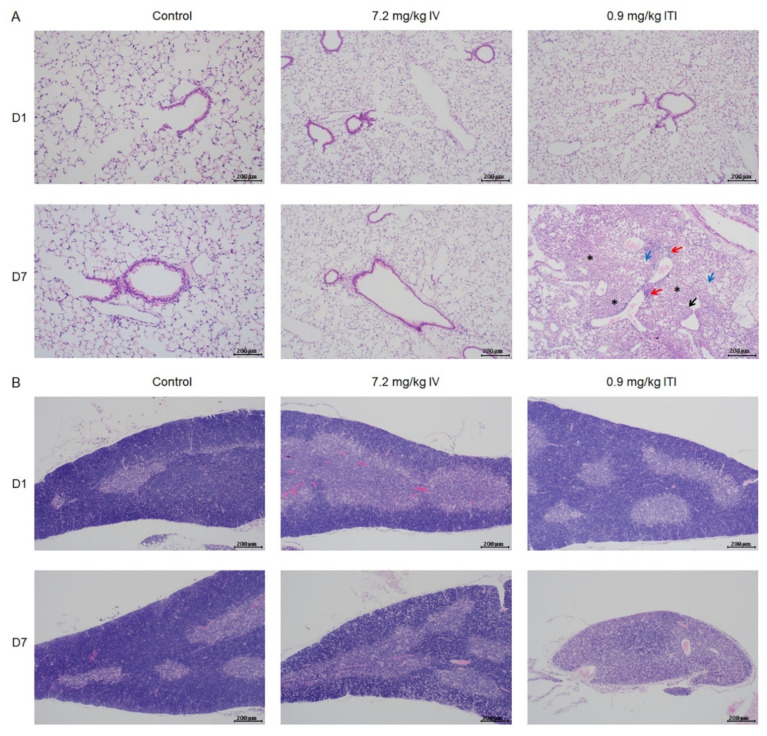

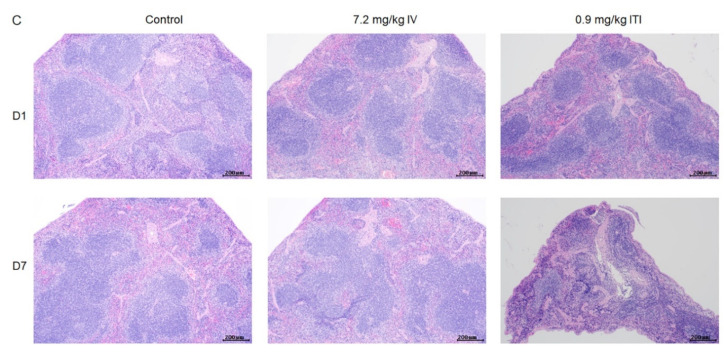
Histopathological examination of PHMG-P-treated mice: Representative photographs of lung (**A**), thymus (**B**), and spleen sections (**C**) are shown. Black arrow indicates degeneration of the bronchiolar epithelium; red arrows indicate the infiltration of inflammatory cells; blue arrows indicate foamy/alveolar macrophages; asterisk indicates fibrosis. Scale bar: 200 µm.

Morphological changes in the lungs, heart, liver, spleen, kidneys, stomach, intestines, thymus, and bone marrow were evaluated. Representative images at each time point are presented in Figure 2 and Supplementary Figure S2. In the 0.9 mg/kg ITI group, abnormal findings—including infiltration of immune cells into the perivascular, peribronchiolar, and interstitial areas of the lungs, degeneration of the bronchiolar epithelia, and fibrotic changes—were detected on day 7. In addition, lymphoid atrophy was observed in the spleen (slight) and thymus (slight to moderate) on days 1 and 7. Marked changes in thymus architecture were detected on day 7 in the 0.9 mg/kg ITI group. A significant loss in cellularity and no medullary–cortex boundary were observed in the 0.9 mg/kg ITI group. No other pathological findings were observed in the liver, kidneys, stomach, intestines, or bone marrow in any of the groups.

### 2.3. Serum Chemistry Analysis

At 4 h after administration of PHMG-P, levels of aspartate aminotransferase (AST) and alanine aminotransferase (ALT), which are indicators of liver damage, increased 1.6–1.7-fold in the 7.2 mg/kg IV group, and 2.0–2.2-fold in the 0.9 mg/kg ITI group (Table 2). Slight increases in BUN, total protein, albumin, ALP, and total cholesterol were observed in the 7.2 mg/kg IV group. Total protein and albumin slightly increased in the 0.9 mg/kg ITI group. Triglyceride (TG) levels were 3.6 times higher in the 7.2 mg/kg IV group and 1.7 times higher in the 0.9 mg/kg ITI group compared to the control group. TG levels returned to normal on day 7 in both groups. On day 1, total cholesterol (TCHO) was slightly increased in the 7.2 mg/kg IV and 0.9 mg/kg ITI groups. These results suggest that PHMG-P alters lipid metabolism. There were no changes in the serum chemistry parameters in the 0.9 mg/kg IV group. The observed changes seem to have been due to the effect of the test material; PHMG-P causes harm immediately after coming into contact with liver cells. Elevated levels of serum AST and ALT reflect hepatocyte integrity (an indicator of liver damage), because these aminotransferases are located in the cytosol or mitochondria, and are released into the serum when the cells are damaged and the plasma membrane is disrupted. The increase in serum AST and ALT levels observed at 4 h after administration shows that these enzymes leak into the serum following damage to hepatocyte membranes. The slight increases in the total protein, albumin, and ALP seem to have been due to the same phenomenon. The levels of these enzymes returned to normal on day 1 after administration.

### 2.4. BAL Total and Differential Cell Counts

**Figure 3:**
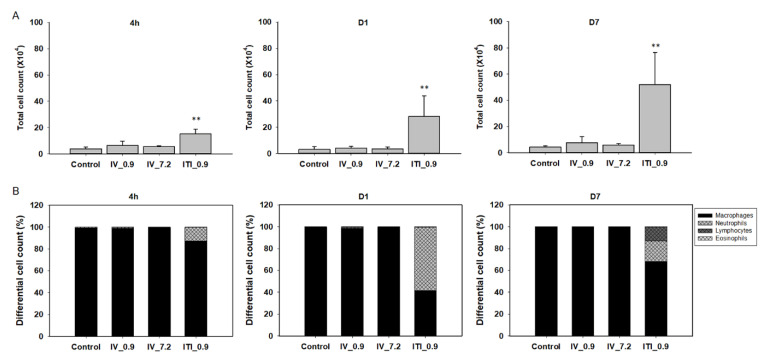

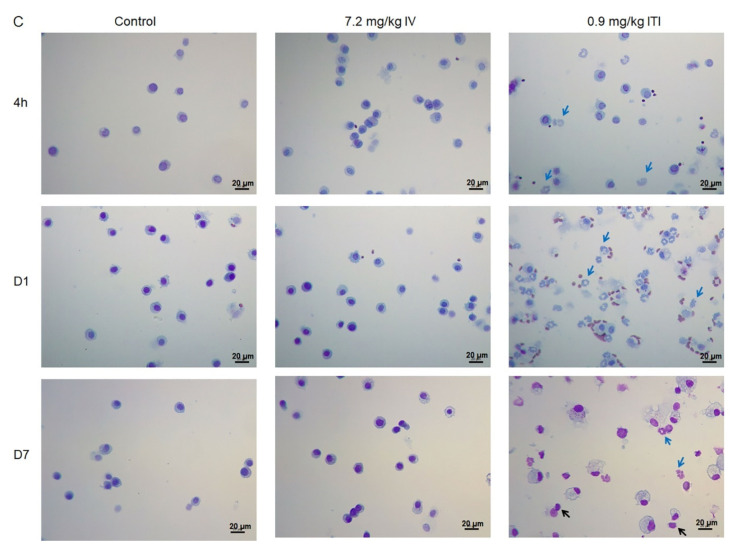
Cellular changes in bronchoalveolar lavage (BAL) of PHMG-P-treated mice: Total cell number in the BAL (**A**), differential cell number in the BAL (**B**), and representative photographs of Diff-Quik-stained BAL cells (**C**). Bars represent the mean ± standard deviation (*n* = 5 per group). Values are significantly different from the control group: ** *p* < 0.01. Blue arrows indicate neutrophils and black arrows indicate lymphocytes. Scale bar: 20 µm.

To investigate the infiltration of immune cells into the lungs, total BAL and differential cell counts were performed. Interestingly, the total cell number in the 0.9 mg/kg ITI group increased by approximately 3.9-fold compared to that of the control group at 4 h after administration of PHMG-P; it increased by 9.3-fold and 11.7-fold on days 1 and 7, respectively. However, there were no changes in the total cell number in the 0.9 mg/kg IV and 7.2 mg/kg IV groups (Figure 3A). Next, the proportion of BAL cells was analyzed (Figure 3B,C); alveolar macrophages were dominant (control group: over 99%). In the 0.9 mg/kg ITI group only, the proportion of neutrophils increased by 12.5%, 57.9%, and 19% at 4 h, on day 1, and on day 7, respectively (Figure 3B). Lymphocytes were detected on day 7 in the 0.9 mg/kg ITI group. No changes in the cell population were observed in the 0.9 mg/kg IV and 7.2 mg/kg IV groups.

### 2.5. Changes in Cytokine Levels in BAL, the Lungs, and the Liver

**Figure 4:**
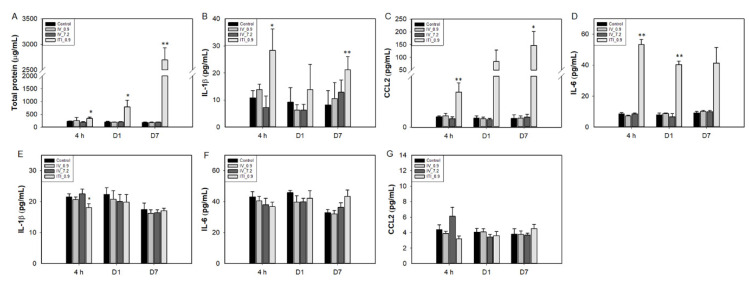
Changes in cytokine levels in the BAL fluid (**A**–**C**), lungs (**D**), and liver lysates (**E**–**G**) of PHMG-P-treated mice. Bars represent the mean ± standard deviation (*n* = 5 per group). Values are significantly different from the control group: * *p* < 0.05, ** *p* < 0.01.

First, BAL fluid and lung lysates were analyzed. Total protein and cytokine levels were not changed in the 0.9 mg/kg IV and 7.2 mg/kg IV groups (Figure 4). In the 0.9 mg/kg ITI group, the total protein increased gradually over time (0.5-, 2.8-, and 13.5-fold vs. control at 4 h and days 1 and 7, respectively). IL-1β and CCL2 production was significantly upregulated at 4 h and day 7 post-exposure in the 0.9 mg/kg ITI group. Interestingly, upregulation of the total protein and cytokines was detected from 4 h after exposure. IL-6 levels increased in the lungs of the 0.9 mg/kg ITI group only.

In the liver, cytokine levels were not changed in any of the treated groups. Levels of the chemokine CCL2 tended to increase in the 7.2 mg/kg IV group at 4 h, but the increase was insignificant due to large interindividual differences, and returned to normal at day 1.

### 2.6. Measurement of HMGB1 and sPLA_2_ in BAL Fluid

**Figure 5:**
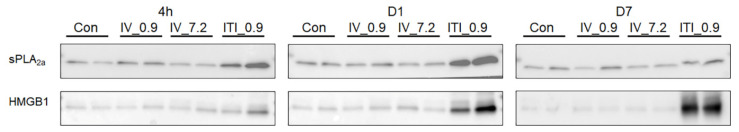
Measurement of HMGB1 and sPLA_2_ in the BAL fluid of PHMG-P-treated mice: Mice were intravenously injected with 0.9 mg/kg (IV_0.9) or 7.2 mg/kg of PHMG-P (IV_7.2), or intratracheally instilled with 0.9 mg/kg of PHMG-P (ITI_0.9). Mice were euthanatized at 4 h and days 1 and 7 after exposure. Protein levels of sPLA_2_ and HMGB1 in the BAL fluid were measured using Western blotting.

First, we measured the expression of HMGB1—a marker of cell necrosis—in BAL fluid. In our previous study, we found that HMGB-1 was released into the media of the human cell lines treated with PHMG-P. In this study, we measured HMGB-1 in the BAL fluid to evaluate whether PHMG-P induces necrosis in the lungs when it is administered via IV injection or ITI. The HMGB-1 levels in BAL fluid were increased at 4 h after instillation, and continued to gradually increase until the end of the experiment, in the 0.9 mg/kg ITI group (Figure 5). Levels of sPLA_2a_—a type of phospholipase A_2_—were increased at 4 h and day 1 after exposure in BAL fluid in the 0.9 mg/kg ITI group, and returned to normal on day 7. However, there was no increase in HMGB-1 or sPLA_2a_ levels in the 0.9 mg/kg IV and 7.2 mg/kg IV groups.

### 2.7. Measurement of mRNA and Protein Expression Levels in the Liver and Lung Tissues

**Figure 6:**
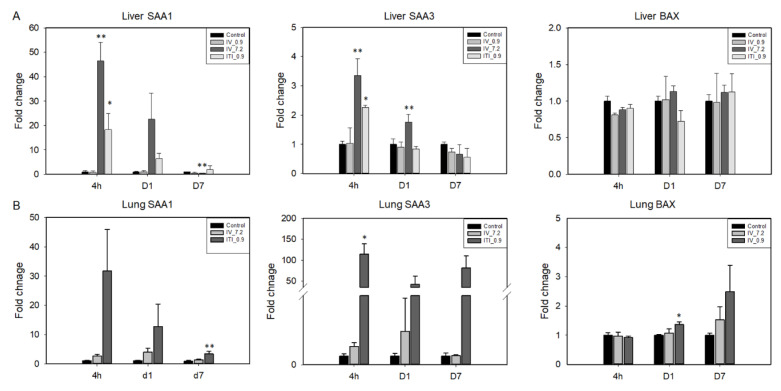
Expression of injury- and apoptosis-related genes was analyzed in the liver and lung tissues using quantitative RT-PCR. mRNA levels of SAA1, SAA3, and BAX in the liver (**A**) and lungs (**B**) are shown. Bars represent the mean ± standard deviation (*n* = 3–4). Values are significantly different from the control group: * *p* < 0.05, ** *p* < 0.01.

To evaluate whether PHMG-P induces damage in the lungs and liver, we measured mRNA levels of serum amyloid A(SAA)1 and SAA3, acute phase proteins, and BAX (Figure 6). In the liver, an increase in the expression levels of SAA1 was detected in the 7.2 mg/kg IV and 0.9 mg/kg ITI groups at 4 h and day 1 after PHMG-P treatment. Liver SAA3 was also significantly upregulated at 4 h in both groups. Interestingly, an increase in the expression levels of SAA1 and SAA3 was not detected in the 0.9 mg/kg IV group. In the case of the lungs, SAA1 and SAA3 levels tended to increase in the 0.9 mg/kg ITI group at 4 h and days 1 and 7; however, there was no statistical significance due to large individual differences.

**Figure 7:**
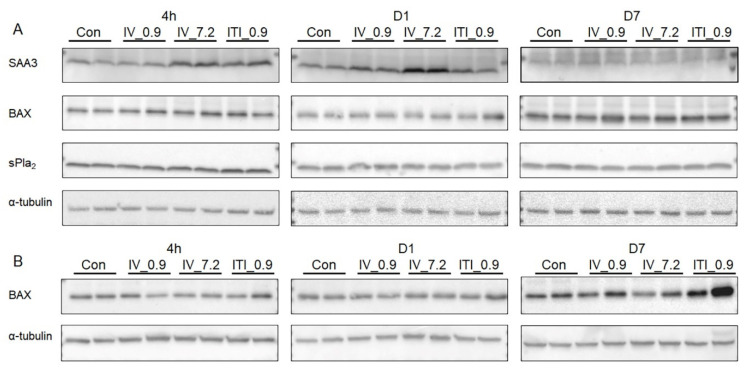
Measurement of protein expression levels in the liver and lung tissues: Protein levels of SAA3, BAX, and sPLA_2_ in the liver lysates (**A**) and BAX in the lung lysates (**B**) were measured using Western blotting. α–Tubulin was used as a loading control.

In the liver, SAA3 protein expression was upregulated at 4 h after treatment in both the 7.2 mg/kg IV and 0.9 mg/kg ITI groups, and increased on day 1 in the 7.2 mg/kg IV group only (Figure 7). To find out whether PHMG-P induces apoptosis, we measured the expression of BAX. BAX expression increased in the lung tissue on day 7, whereas there was no change in the liver in any of the groups. In addition, the expression of sPLA_2_ in the liver did not change in the liver of any of the groups.

### 2.8. Zebrafish Assay

**Figure 8:**
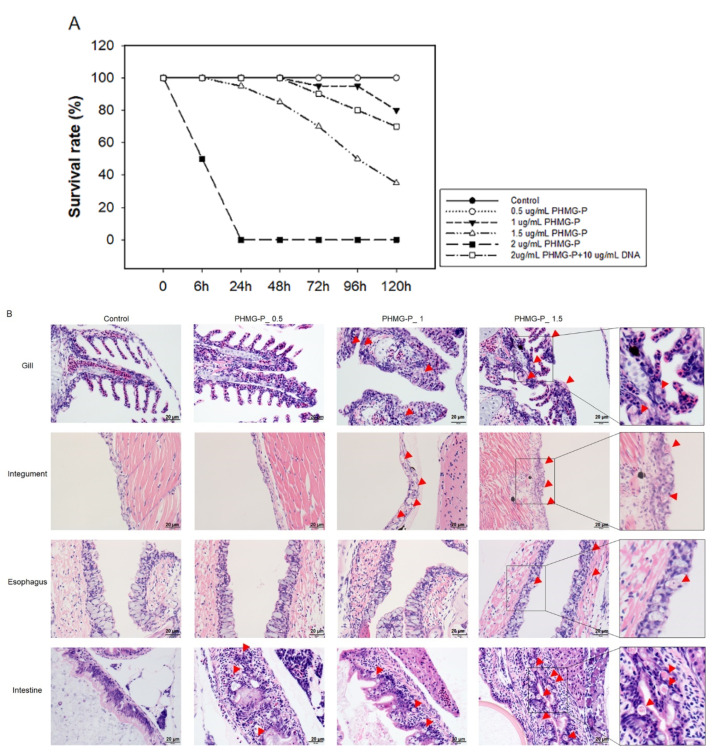
Survival rates and histopathological examination of PHMG-P-treated zebrafish: Four-week-old zebrafish were exposed to 0.5, 1, 1.5, or 2 μg/mL PHMG-P for 5 days. The mortality was observed at a designated time (**A**). Survival rates are expressed as the mean ± standard deviation (*n* = 20). Representative photographs (**B**) of PHMG-P-treated zebrafish. The red triangle indicates necrosis of the epithelial cell. Scale bar: 20 µm.

We used zebrafish, which have been used for toxicological studies [18,19], to further examine the effects of PHMG-P on survival and pathogenesis. As shown in Figure 8A, the survival rate of the zebrafish in the PHMG-P-treated groups decreased in a dose-dependent manner. Although all the zebrafish treated with 2 μg/mL PHMG-P died on day 1 after exposure, the group co-treated with 2 μg/mL PHMG-P and 10 μg/mL DNA showed delayed mortality and a lower overall mortality rate (10% on day 3, 20% at day 4, and 30% on day 5).

In the histopathological analysis, necrosis of the epithelial cells was observed in the gills, integuments, esophagus, and intestines (Figure 8B). No pathological abnormality was detected in any other organs, such as the kidneys or liver. PGH—another guanidine family biocide—also showed necrosis of epithelial cells of the same organs (Supplementary Figure S3).

### 2.9. PHMG-P Induced Cytotoxicity at Various Serum Concentrations

**Figure 9:**
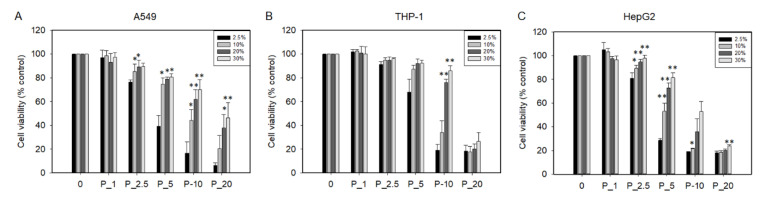
Cytotoxicity of A549 (**A**), THP-1 (**B**), and HepG2 cells (**C**) exposed to PHMG-P in the cell culture media supplemented with 2.5%, 10%, 20%, or 30% fetal bovine serum (FBS). Cell viability was measured using CCK-8 reagent. Cell viability is shown as a percentage of control. Data are expressed as the mean ± standard deviation of three separate experiments. Values are significant compared to cell viability of 2.5% FBS at each concentration of PHMG-P; * *p* < 0.05, ** *p* < 0.01.

PHMG-P induced cytotoxicity in A549, THP-1, and HepG2 cells, in a dose-dependent manner. To verify the effect of serum on PHMG-P-induced cytotoxicity, various concentrations of PHMG-P were applied in culture media containing 2.5%, 10%, 20%, or 30% FBS. As shown in Figure 9, cell viability increased with increasing FBS concentration. However, when cells were treated with caffeine or cyclophosphamide—negative control materials—there was no significant increase in the cell viability with increased serum concentration, although a slight increase in the cell viability was seen in cyclophosphamide-treated THP-1 cells in culture media containing 20% and 30% FBS (Supplementary Figure S4).

## 3. Discussion

Several studies have discussed the toxic mechanisms of PHMG-P, which include reactive oxygen species (ROS) production, cytokine production through NF-κB activation, and impairment of the tight junction by impairing the F-actin and influx of Ca2^+^ via P2X purinoreceptor 7 [20,21]. Our previous study showed that PHMG-P disrupts the eukaryotic cell plasma membranes through ionic interaction between the cations of PHMG-P and anions in the cell membrane [13]. PHMG-P induces cell death, HMGB1 is released into the culture medium, and cytokine and ROS are produced, whereas co-treatment of PHMG-P and anionic materials such as DNA or poly-L-glutamic acids ameliorates these molecular events. Hong et al. [22] used atomic force microscopy to show that cationic materials perforate holes in the lipid bilayers. Recently, Paliienko et al. [23] revealed that polyhexamethylene guanidine hydrochloride formed weakly cation-selective transmembrane pores in phospholipid bilayers by measuring transmembrane current. Membrane damage caused by cationic materials leads to increased permeability of lipid bilayer membranes and leakage of cytosolic enzymes out of the cells. However, this increased membrane permeability can be reversed by the removal of cationic materials [22]. Small wounds in the plasma membrane can be repaired in a short period (seconds to minutes) [24,25,26]. Spontaneous membrane resealing can be accomplished by Ca^2+^-dependent exocytosis of the internal membranes and the fusion of the internal membranes with the plasma membrane [24,25]. Excessive membrane damage that cannot be repaired by the cells is likely to induce cell death.

Based on the above PHMG-P cytotoxicity mechanism, we hypothesized that the toxicity of PHMG-P is highly dependent on the route of administration, because PHMG-P needs to come into direct contact with cells in order to have its harmful effects. Some routes of administration are less likely than others to result in direct cellular interactions with the toxic substance. For example, various kinds of serum proteins (3.5–5.0 g/dL in humans; 4.0–5.1 in rodents) [27,28] in intravenous injection, miscellaneous food in the stomach following oral gavage, and dermal exposure of the stratum corneum can block direct ionic interactions between PHMG-P and the cell membranes. Therefore, the cationic nature of PHMG-P might induce more severe and lethal effects via inhalation than by other routes because the lung cells can come into direct contact with the PHMG-P in the lining lung fluid (total protein: 0.0075 g/dL in humans; 0.003 g/dL in mice) [29], with or without less hindrance of ionic interaction. Actually, the LD_50_ (median lethal dose) of PHMG-P is 450 mg/kg or 600 mg/kg when given orally in mice and rats, respectively [30,31], whereas all mice died when 3 mg/kg PHMG-P was given intratracheally in our previous study [32]. The acute dermal LD_50_ of PHMG-P is over 2000 mg/kg (NICNAS, 2003). These results show that PHMG-P toxicity is more severe with inhalation exposure than via the other exposure routes. Currently, there are no toxicity data on intravenously administered PHMG-P. In this study, we chose IV injection because test materials administrated intravenously are highly absorbed in the body, and rapidly circulate systemically without passing through the liver, unlike those inhaled. We compared the toxicity between PHMG-P administered through intratracheal instillation and intravenous injection.

First, no toxicity was detected when 0.9 mg/kg PHMG-P was administered intravenously. IV injection of 7.2 mg/kg PHMG-P resulted in loss of body weight and decreases in liver, spleen, and thymus weights; the same effects were observed in mice that were intratracheally instilled with 0.9 mg/kg PHMG-P (ITI group). Interestingly, IV injection of 7.2 mg/kg PHMG-P did not have any toxic effects on the lungs, such as increases in BAL cell number, cytokine production, or pathological findings, whereas 0.9 mg/kg PHMG-P ITI induced a fibroinflammatory response in the lungs.

To verify whether PHMG-P induces cell death through direct contact, cell viability was measured by testing media containing various concentrations of FBS. Increased FBS concentration ameliorated cell viability in A549, THP-1, and HepG2 cells. Chemicals absorbed into the blood are bound to plasma proteins; the main proteins in human plasma are serum albumin and alpha-1-acid glycoprotein [33]. Hydrophobic and acidic chemicals preferentially bind to albumin, which is the most abundant plasma protein, while basic chemicals bind mainly to the alpha-1-acid glycoprotein [34,35]. The guanidine group of PHMG-P is a strong organic base, so it is thought to bind mainly to alpha-1-acid glycoprotein and other anionic materials. Serum proteins bind to PHMG-P and hinder its interaction with the cell membrane. Serum proteins are known to form a protein coating by binding to nanoparticles, forming a structure called a protein corona. Likewise, PHMG-P seems to form a protein corona, which reduces its toxicity. The results of the zebrafish assay support this hypothesis; co-treatment of PHMG-P with DNA—an anionic material that neutralizes the cation of PHMG-P—dramatically ameliorated the decline in survival of the PHMG-P-exposed zebrafish. In addition, the abnormal histopathological findings induced by PHMG-P were detected in the gills, integuments, stomach, and intestines, all of which came into direct contact with PHMG-P. Based on these results, we can conclude that the route of administration is an essential risk factor in PHMG-P-induced toxicity.

Bewersdorf et al. reported that nanoparticles bound to plasma proteins tend to be cleared from the body rapidly by the reticuloendothelial system [36]. The PHMG-P particle size is known to be smaller than 100 nm [1,2], so it is likely to behave like a nanoparticle in the blood. Based on this, we hypothesized that PHMG-P administered through IV might be cleared out of the body faster than that given through ITI.

Next, we evaluated whether PHMG-P is toxic to tissues other than the lungs, which depends on how much PHMG-P can be distributed to the body. Histopathological examination revealed no toxicity in the liver, kidneys, heart, stomach, intestines, or bone marrow in any of the treated groups. However, there have been some reports on liver toxicity induced by PHMG-P. Ostapenko et al. [37] reported that patients who consumed illegally manufactured vodka composed of ethanol (≈93%), diethyl phthalate, and 0.1–0.14% PHMG were hospitalized at high rates, and the levels of their hepatic enzymes—such as ALT, AST, and GGT—increased [37]. Shim et al. (2018) reported that radio-labeled PHMG-P was retained in the lungs (approximately 45%) and liver (approximately 7–8%) for 7 days when given via ITI, whereas orally ingested PHMG-P was not detected in the lungs or liver [38]. Rats exposed to high doses of PHMG-P (25 mg/m^3^) using a whole-body chamber for 2 weeks also showed elevated ALT and AST levels, and slight decreases in the liver weight on the next day after exposure [39]. In this study, we found that a single dose of PHMG-P through 0.9 mg/kg ITI or 7.2 mg/kg IV leads to elevations in liver enzymes and TG concentration at 4 h after administration, while there were no changes in the liver enzyme levels in the 0.9 mg/kg IV group. The increased levels of AST and ALT observed in the serum revealed that PHMG-P induces hepatocyte damage. Serum AST and ALT elevation reflects hepatocyte damage, because these aminotransferases are located in the cytosol or mitochondria, and are released into the serum when cells are damaged and the plasma membrane is disrupted [40]. In both groups, the levels of these enzymes returned to normal on day 1. This indicates that PHMG-P disturbs hepatocyte membranes immediately after contact, allowing these enzymes in the cytosol to leak into the blood. However, PHMG-P-induced damage in the plasma membrane seems to be repaired rapidly, which might be because the PHMG-P bound to plasma proteins is rapidly cleared from the body, as mentioned by Bewersdorf et al. [36].

SAA is an acute-phase protein that is highly upregulated during inflammation or injury; it modulates the innate and adaptive immune responses [41], and has been shown to promote chemotaxis of human monocytes and murine macrophages through TLR2, and of neutrophils through formyl peptide receptor (FPR)-like 1/FPR2 [42]. The liver is the main site of SAA1 synthesis, while SAA3 is expressed at extrahepatic sites [43]. In this study, hepatic SAA1 and SAA3 mRNA increased in both the 7.2 mg/kg IV and 0.9 mg/kg ITI groups at 4 h and/or day 1 after administration, but not in the 0.9 mg/kg IV group. However, SAA1 and SAA3 also returned to normal at day 1 or day 7. Considering these results, a high dose of PHMG-P IV injection and a low dose of PHMG-P intratracheal instillation damaged the liver cells; however, these toxic effects rapidly disappeared. Interestingly, 0.9 mg/kg ITI evoked liver damage that was similar to that seen in the 7.2 mg/kg IV group. When given through ITI, PHMG-P might reach the liver cells through the bloodstream, like the IV route. How can a low ITI dose of PHMG-P induce a similar degree of liver damage to that of 7.2 mg/kg IV injection? It could be because the production of inflammatory mediators—such as prostaglandins, cytokines, chemokines, or acute phase proteins such as SAA and fibrinogen—is induced in the lungs and distributed in the other organs, such as the liver, via blood. As shown in Figure 4, total proteins, IL-1β, and IL-6 were greatly increased in BAL fluid at 4 h after treatment. mRNA expression levels of lung SAA1 and SAA3 were also upregulated in the 0.9 mg/kg ITI group. IL-1, TNF-α, and IL-6 are known to induce SAA [43]. Saber et al. [44] reported that intratracheal instillation of nanomaterials resulted in a marked increase in SAA3 in the serum, as well as in the lungs. These results support the hypothesis that SAA3 or proinflammatory cytokines produced in the lungs were transferred to the liver via the blood, resulting in elevated liver SAA1 or SAA3 levels; further research on this hypothesis should be carried out. In addition, research is needed in order to determine whether chronic exposure to PHMG-P via IV injection or other routes of exposure can induce damage in tissues other than the lungs.

PLA_2_ is involved in membrane phospholipid degradation and cellular damage, and may be particularly active at sites with structural defects [45]. Researchers have reported that healthy cells are resistant to PLA_2_, but cells with damaged membranes or dying cells are susceptible to PLA_2_ activity [46,47]. This increased susceptibility to hydrolysis is due to alterations in the structure and dynamics of the plasma cell membrane, such as increased lipid spacing, decreased lipid order, and increased exposure of anionic phospholipids—for example, phosphatidylserine—on the outer face of the cell membrane [48,49]. The compromised plasma membrane induced by PHMG-P seems to increase the cells’ vulnerability to PLA_2_ attack. In this study, elevated sPLA_2a_ and total protein levels were seen in the BAL fluid of the ITI group only, supporting the suggestion that PHMG-P induces membrane damage with increased membrane permeability, and that direct contact between PHMG-P and cell membranes is a key factor in PHMG-induced toxicity.

In this study, we found that direct exposure of the lungs to PHMG-P produced a higher level of toxicity than any of the other exposure routes. Notably, there was no lung injury following IV injection, even though some IV-treated mice received a dose that was eight times higher than that of the ITI group. The 7.2 mg/kg IV and 0.9 mg/kg ITI groups showed elevated liver-related enzyme levels and triglyceride concentrations, but these levels returned to normal levels on day 1 after treatment or afterward. No histopathological abnormalities were seen in the heart, liver, kidneys, intestines, or bone marrow. We confirmed that PHMG-P induces toxic effects by disrupting the membrane integrity through ionic interactions, and that some routes of administration—for example, IV injection or oral administration—are less likely to result in direct interactions between cellular plasma membranes and PHMG-P, therefore leading to minimal toxicity. In addition, this study confirmed that the disruption of membrane integrity is a molecular initiating event of adverse effects induced by cationic materials, which are much more harmful when inhaled than when taken in through other routes. Therefore, the adverse effects of cationic chemicals should be carefully assessed according to the route of administration.

## 4. Materials and Methods

### 4.1. Materials

PHMG-P (25%) and saline were purchased from BOC Sciences (Shirley, NY, USA) and Daihan Pharmaceutical Co. (Ansan, Korea).

### 4.2. Animal Experiment

Seven-week-old male C57BL/6 mice (Orient Bio Inc., Seongnam, Korea) were housed in an environmentally controlled animal room at a temperature of 22 ± 3 °C, relative humidity of 50 ± 10%, and air ventilation of 10–20 times/h, with a 12 h light/dark cycle. All animals had access to a sterile rodent pellet diet (PM Nutrition International, Richmond, USA) and UV-irradiated (Steritron SX-1; Daeyoung, Inc. Korea) tap water ad libitum. All the experiments were approved by the Institutional Animal Care and Use Committee of Korea Institute of Toxicology (approval number: 2008-0268), and conducted according to the guidelines of the Association for Assessment and Accreditation of Laboratory Animal Care International.

The mice were randomly divided into four groups using Pristima (Version 7.4; Xybion Medical Systems Corporation, Morris Plains, NJ, USA). A master PHMG-P solution (25%) was diluted to 0.9 and 7.2 mg/kg (5 mL/kg) with saline, and was then injected intravenously through the mice’s tail veins, while 0.9 mg/kg PHMG-P (50 μL/mouse) was instilled intratracheally after the mice were anesthetized with isoflurane. The control group was administered saline intravenously. Body weight was measured three days before dosing and on days 2, 3, 5, and 8. At 4 h and days 1 and 7 after administration, mice were euthanatized with an overdose of isoflurane. The spleen, thymus, liver, heart, kidneys, and lungs were weighed. The spleen, thymus, liver, heart, kidneys, stomach, intestines, and left lung were fixed in 10% neutral buffered formalin. The right lungs and liver were snap-frozen for further evaluation.

The PHMG-P dose was chosen on the basis of a preliminary study, in which ITI of 0.9 mg/kg PHMG-P induced pulmonary fibrosis of a moderate degree with minimal mortality [32]. We set the doses for IV administration at 0.9 mg/kg and 7.2 mg/kg, at which the mice showed subdued behavior and hunched posture.

### 4.3. Bronchoalveolar Lavage

Five mice per group underwent bronchoalveolar lavage (BAL) on each necropsy day. After the mice were anesthetized with isoflurane, blood was drawn from the abdominal aorta. Then, the left lung was ligated and the right lung was lavaged with cold phosphate-buffered saline (PBS, 0.7 mL) through a tracheal cannula. The BAL fluid was centrifuged at 2000 rpm for 5 min, and the supernatant was stored at −70 °C for further analysis. The total number of BAL cells was counted using NucleoCounter (NC-250, ChemoMetec, Gydevang, Denmark). For differential cell counts, BAL cells were cytospun at 800 rpm for 10 min. Slides containing these cells were prepared, then dried and fixed in methanol. The slides were put in Sol I. solution of Diff-Quik solution (Dade Diagnostics, Aguada, Puerto Rico) and washed, then put in Sol II. Cover slides were put on the slides. Cells were counted at 200 cells per slide for differential counting under a microscope.

### 4.4. Histopathological Examination

The lungs, heart, liver, kidneys, spleen, thymus, stomach, intestines, and bone marrow were fixed in 10% neutral formalin buffer. Bone marrow (femur) was transferred to 5% formic acid and decalcified for 5 days. For histological examination, sequential 4 µm thick paraffin sections were cut from each embedded tissue and stained with hematoxylin and eosin (H&E) and Masson’s trichrome (lungs only). Tissue slides were analyzed under light microscopy (Olympus BX53, Tokyo, Japan). The severity grading of inflammation and fibrosis was scored on a subjective scale of 0–5 (0 (normal) = no presence of lesion; 1 (minimal) = presence of lesions involving < 20% of the tissues; 2 (slight) = lesions involving 20–40% of the tissue; 3 (moderate) = lesions involving 40–60% of the tissue; 4 (marked) = lesions involving 60–80% of the tissue; and 5 (severe) = lesions involving > 80% of the tissue).

### 4.5. Serum Chemistry

Blood was collected in serum separation tubes, and serum was separated by centrifugation at 3000 rpm for 10 min. Serum was analyzed using a Toshiba 200FR NEO (Toshiba Co., Tokyo, Japan).

### 4.6. Cytokine Measurement in BAL Fluid, the Lungs, and the Liver

The frozen livers and lungs were homogenized using a homogenizer (IKA, Staufen, Germany) in lysis buffer (Thermo Scientific, Waltham, MA, USA) on ice and incubated at 4 °C for 30 min. The lysates were centrifuged at 13,000 rpm for 30 min at 4 °C. The supernatants were collected. Interleukin-1β (IL-1β), interleukin-6 (IL-6), and CCL-2 (liver lysate only) in BAL fluid and tissue lysates were quantified using commercial ELISA kits (R&D Systems, Minneapolis, MN, USA), according to the manufacturer’s protocol. The total protein levels of the BAL fluid and tissue lysates were quantified using the BCA Protein Assay Kit (Sigma-Aldrich, St. Louis, MO, USA). The cytokine levels of the tissue lysates were normalized using total protein.

### 4.7. Quantitative Real-Time PCR

Total RNA was extracted from mice’s lungs and livers using the RNeasy Mini Kit (Qiagen, Hilden, Germany), according to the manufacturer’s instructions. A NanoDrop ND-1000 spectrophotometer (NanoDrop Technologies, Wilmington, DE, USA) was used for measuring the RNA purity and concentration. RNA samples with A260/280 and A260/230 ratios of > 1.8 were used for cDNA synthesis. One microgram of the total RNA was reverse-transcribed to cDNA using the GoScript^TM^ Reverse Transcription System (Promega, Madison, WI, USA). The sequences of the primers were as follows: SAA1: sense, 5′-CTCCTATTAGCTCAGTAGGTTGTG-3′, antisense, 5′-CACTTCCAAGTTTCTGTTTATTACCC-3′; SAA3: sense, 5′-GCCTGGGCTGCTAAAGTCAT-3′, antisense, 5′-TGCTCCATGTCCCGTGAAC-3′; BAX: sense, 5′- GGGCCCACCAGCTCTGA-3′, antisense, 5′-TGGATGAAACCCTGTAGCAAAA-3′; Hprt: sense, 5′- GTACTTCAGGGATTTGAATCACGTT-3′, antisense, 5′-ATTTGCAGATTCAACTTGCGCT-3′. The specificity of the primers and the PCR efficiency were validated with the slope of the standard curve, the melting curve, and agarose gel electrophoresis. PCR reactions were performed using SYBR Green PCR Master Mix (Applied Biosystems, Woolston, Warrington, UK). The relative expression levels were calculated using the comparative threshold cycle (delta-delta CT) method, and were presented as fold-changes (Bustin et al., 2009). Hprt was used as the reference gene. All reactions were carried out in three biological replicates and two technical replicates.

### 4.8. Western Blot Analysis

We measured the expression of HMGB1 and sPLA_2_ in BAL fluid, sPLA_2_, BAX, and SAA3 in the liver, and BAX in the lung tissues. An equal amount of BAL fluid and equal quantities of tissue lysates were resolved using SDS–PAGE (Bio-Rad, CA, USA). Each sample was transferred to a PVDF membrane (Bio-Rad, CA, USA). The membranes were incubated with 5% non-fat milk in Tris-buffered saline with 0.05% Tween 20 (TBST) for 1 h at room temperature, and incubated with primary antibodies overnight at 4°C with shaking. After washing three times with TBST, the membranes were incubated for 45 min with species-appropriate horseradish-peroxidase-conjugated secondary antibodies. The protein bands were detected using chemiluminescent Western blotting detection reagents (Thermo, Rockford, IL, USA). Anti-rabbit HMGB1 antibody (Abcam, Cambridge, UK), anti-rabbit sPLA_2a_ antibody (Abcam, Cambridge, UK), anti-mouse β-actin (Santa Cruz, CA, USA), and anti-rabbit α-tubulin (Cell Signaling, MA, USA) were used for detection of the corresponding proteins.

### 4.9. Cell Culture

We performed cell experiments to simulate IV injection of PHMG-P. A549 (human lung epithelial cells) and THP-1 (human monocyte cells) cells were purchased from the Korean Cell Bank. The HepG2 line (human liver cells) was obtained from ATCC. A549 and THP-1 were suspended in RPMI 1640, supplemented with 10% fetal bovine serum (FBS) (Thermo Scientific) and penicillin/streptomycin. HepG2 was grown in DMEM supplemented with 10% FBS (Thermo Scientific) and penicillin/streptomycin. A549 (0.8 × 10^5^ cells per well), THP-1 (1 × 10^5^ cells per well), and HepG2 (1 × 10^5^ cells per well) cells were seeded in 96-well plates. The next day, the media were removed and replenished with fresh culture media supplemented with 2.5%, 10%, 20%, or 30% FBS. Then, PHMG-P was added to each well. After 24 h, cell viability was measured using CCK-8 reagent (Dojindo, Kumamoto, Japan), according to the manufacturer’s protocol. Ten microliters of CCK-8 was added to each well, and the plates were incubated for 2 h. Optical density (OD) was measured at 450 nm using a SpectraMax Plus 384 microplate reader (Molecular Device, San Jose, CA, USA). Cell viability was expressed as a percentage of control.

### 4.10. Zebrafish Assay

Wild-type zebrafish (Danio rerio) were raised and maintained on a 14:10 h light:dark cycle at 28 °C. Healthy 4-week-old zebrafish were collected and 120 zebrafish were randomly selected and placed in groups of 6. The zebrafish were treated with 0.5, 1, 1.5, or 2 ug/mL PHMG-P. To verify the mechanism of toxicity of PHMG-P, another group was treated with 10 μg/mL DNA and 2 μg/mL PHMG-P. During the test, half of the water with fresh chemical solution was replenished every day. The dose was chosen from a previous 96-hour zebrafish embryo acute toxicity test (data not shown). The exposed zebrafish were observed every day for 5 days. On day 5, zebrafish were fixed in 4% paraformaldehyde and incubated at 4 °C for 48 h. After a decalcification with 5% formic acid solution for a day, they were washed with tap water. Whole-body specimens were dehydrated and embedded in paraffin (Leica HistoCore Arcadia H). Three sequential 2.5 µm thick paraffin sections were cut with a microtome (Leica RM2255) and stained with H&E. The slides were examined under light microscopy (Nikon Eclipse E600). All of the experiments were approved by the Institutional Animal Care and Use Committee of Wonkwang University (approval number: WKU21-65).

### 4.11. Data Analysis

One-way analysis of variance with Tukey’s post-hoc test or Kruskal–Wallis nonparametric analysis with Dunn’s multiple comparison test was used to determine differences between groups (SPSS Ver15.0.0, SPSS Inc., Chicago, IL, USA). A *p*-value of less than 0.05 was considered statistically significant. All data are presented as means ± standard deviations.

## 5. Conclusions

PHMG-P causes fatal lung damage when inhaled. However, it shows lower toxicity when given via routes other than inhalation. In this study, IV injection of 7.2 mg/kg PHMG-P did not cause any lung damage, whereas ITI of 0.9 mg/kg PHMG-P led to fibroinflammatory changes in the lungs. PHMG-P induces toxic effects by disrupting the membrane integrity through ionic interactions. In the case of IV injection, various kinds of serum proteins readily bind to PHMG-P and hinder the interaction between PHMG-P and cellular membranes, thus resulting in low toxicity. PHMG-P-treated zebrafish showed necrosis of epithelial cells in the gills, esophagus, and intestines, which came into direct contact with PHMG-P. On the other hand, histopathological lesions were not detected in the esophagus or intestines in PHMG-P-treated mice when given through either IV or ITI. These results show that direct contact between PHMG-P and cell membranes is a key factor in PHMG-P-induced toxicity.

## Figures and Tables

**Table 1:** Mean body weight of PHMG-P-treated mice.

Day	Control	0.9 mg/kg IV	7.2 mg/kg IV	0.9 mg/kg ITI
−3	21.80 ± 0.75 ^a^	22.04 ± 0.63	21.95 ± 0.80	21.80 ± 0.68
1	22.68 ± 0.86	23.19 ± 0.76	20.32 ± 1.12 **	19.49 ± 0.71 **
2	22.02 ± 0.85	22.48 ± 0.71	19.87 ± 1.17 **	17.36 ± 0.65 **
4	22.71 ± 0.90	23.53 ± 0.80	20.16 ± 1.29 **	17.07 ± 0.78 **
7	23.04 ± 0.97	24.13 ± 0.83	20.66 ± 0.97 **	18.64 ± 1.60 **

^a^ Data are expressed as mean ± standard deviation. Values are significantly different from the control group: ** *p* < 0.01. The number of mice per group was 27 at day −3, 18 at day 1, and 9 from day 2 through day 7.

**Table 2:** Changes in serum chemistry following exposure to PHMG-P.

4 h	Control	0.9 mg/kg IV	7.2 mg/kg IV	0.9 mg/kg ITI
BUN ^a^ (mg/dL)	22.80 ± 1.74	20.30 ± 2.82	26.30 ± 3.11 *	23.20 ± 1.67
CREA (mg/dL)	0.28 ± 0.04	0.29 ± 0.02	0.29 ± 0.03	0.23 ± 0.02 *
TP (g/dL)	5.11 ± 0.18	4.87 ± 0.22	5.85 ± 0.28 **	5.54 ± 0.38 *
ALB (g/dL)	3.31 ± 0.11	3.15 ± 0.19	3.67 ± 0.16 **	3.52 ± 0.19 *
AST (IU/L)	55.60 ± 15.73	55.20 ± 4.77	92.10 ± 8.20 **	108.90 ± 31.72 **
ALT (IU/L)	28.80 ± 5.30	29.20 ± 3.7	45.20 ± 11.35 *	62.70 ± 34.23
GGT (IU/L)	1.85 ± 0.27	1.82 ± 0.35	2.06 ± 0.37	2.09 ± 0.59
ALP (IU/L)	485.10 ± 36.46	491.90 ± 39.36	541.50 ± 45.88 *	483.90 ± 49.16
TCHO (mg/dL)	84.10 ± 4.59	82.90 ± 5.99	98.10 ± 7.82**	88.80 ± 5.36
TG (mg/dL)	46.00 ± 7.12	66.80 ± 20.17	164.70 ± 94.69 *	77.70 ± 13.22 **
**D1**	**Control**	**0.9 mg/kg IV**	**7.2 mg/kg IV**	**0.9 mg/kg ITI**
BUN (mg/dL)	27.20 ± 3.98	28.60 ± 4.30	20.20 ± 2.68 **	21.50 ± 2.46 *
CREA (mg/dL)	0.33 ± 0.04	0.34 ± 0.04	0.30 ± 0.03	0.28 ± 0.02
TP (g/dL)	5.34 ± 0.33	5.30 ± 0.30	5.60 ± 0.25	5.51 ± 0.30
ALB (g/dL)	3.45 ± 0.16	3.42 ± 0.12	3.63 ± 0.19	3.59 ± 0.16
AST (IU/L)	48.20 ± 6.31	45.40 ± 4.13	53.8 ± 5.94	52.7 ± 4.79
ALT (IU/L)	28.10 ± 4.39	28.90 ± 3.43	23.30 ± 1.45 *	23.10 ± 3.50 *
GGT (IU/L)	1.96 ± 0.14	1.93 ± 0.40	2.08 ± 0.29	1.92 ± 0.34
ALP (IU/L)	485.30 ± 57.82	534.80 ± 51.46	457.60 ± 52.09	480.80 ± 51.26
TCHO (mg/dL)	85.40 ± 4.90	88.60 ± 5.39	101.60 ± 9.48 **	95.80 ± 4.76 **
TG (mg/dL)	66.50 ± 15.83	64.80 ± 13.22	46.50 ± 10.52 *	57.00 ± 19.31
**D7**	**Control**	**0.9 mg/kg IV**	**7.2 mg/kg IV**	**0.9 mg/kg ITI**
BUN (mg/dL)	30.00 ± 4.13	29.80 ± 3.66	35.30 ± 6.82	30.30 ± 4.24
CREA (mg/dL)	0.31 ± 0.04	0.31 ± 0.03	0.32 ± 0.03	0.29 ± 0.03
TP (g/dL)	5.53 ± 0.15	5.59 ± 0.29	5.51 ± 0.30	5.51 ± 0.34
ALB (g/dL)	3.41 ± 0.09	3.39 ± 0.15	3.47 ± 0.18	3.49 ± 0.18
AST (IU/L)	48.20 ± 2.77	47.40 ± 4.58	57.40 ± 6.51 *	61.30 ± 10.44 **
ALT (IU/L)	31.10 ± 1.78	32.00 ± 5.33	31.60 ± 3.60	38.60 ± 8.29
GGT (IU/L)	1.66 ± 0.32	1.77 ± 0.21	1.91 ± 0.32	1.78 ± 0.19
ALP (IU/L)	426.90 ± 51.04	450.80 ± 49.71	405.80 ± 30.36	376.90 ± 40.01
TCHO (mg/dL)	84.89 ± 7.72	83.22 ± 6.40	93.33 ± 6.89	96.11 ± 8.59 *
TG (mg/dL)	94.47 ± 24.30	72.33 ± 12.31	90.33 ± 31.97	80.58 ± 22.88

^a^ BUN: blood urea nitrogen; CREA: creatinine; TP: total protein; ALB: albumin; AST: aspartate aminotransferase; ALT: alanine aminotransferase; GGT: gamma glutamyl transpeptidase; ALP: alkaline phosphatase; TCHO: total cholesterol; TG: triglyceride. Data are expressed as mean ± standard deviation (*n* = 9 per group). Values are significantly different from the control group: * *p* < 0.05, ** *p* < 0.01.

## Data Availability

Data sharing not applicable.

## Supplementary Materials

The following supporting information can be downloaded at: https://www.mdpi.com/article/10.3390/ijms23063289/s1.
