# Supplementary material for: Disruption of Membrane Integrity as a Molecular Initiating Event Determines the Toxicity of Polyhexamethylene Guanidine Phosphate Depending on the Routes of Exposure
Source: Int J Mol Sci. 2022 Mar 18;23(6):3289. doi: 10.3390/ijms23063289 (PMC8955148; doi:10.3390/ijms23063289)
Supplement: Supplementary file 1 [file ijms-23-03289-s001.zip › ijms-1624904-supplementary.pdf]

Supplementary material

Disruption of Membrane Integrity as a Molecular Initiating Event Determines the Toxicity of Polyhexamethylene Guanidine Phosphate Depending on the Routes of Exposure

Jeongah Song [1,*], Kyung-Jin Jung [2], Mi-Jin Yang [3], Woojin Kim [4], Byoung-Seok Lee [4], Seong-Kyu Choe [5,6], Seong-Jin Kim [6] and Jeong-Ho Hwang [1]

[1] Animal Model Research Group, Korea Institute of Toxicology, Jeongeup 56212, Korea; jeongho.hwang@kitox.re.kr
[2] Bioanalytical and Immunoanalytical Research Group, Korea Institute of Toxicology, Daejeon 34114, Korea; jungk@kitox.re.kr
[3] Jeonbuk Pathology Research Group, Korea Institute of Toxicology, Jeongeup 56212, Korea; mjyang@kitox.re.kr
[4] Toxicologic Pathology Research Group, Korea Institute of Toxicology, Daejeon 34114, Korea; woojinkim@kitox.re.kr (W.K.); bslee@kitox.re.kr (B.-S.L.)
[5] Department of Microbiology, Wonkwang University School of Medicine, Iksan 54538, Korea; seongkyu642@wku.ac.kr
[6] Department of Biomedical Science, Graduate School, Wonkwang University, Iksan 54538, Korea; mbunker1.21@gmail.com
* Correspondence: jasong@kitox.re.kr; Tel.: +82-63-850-8553

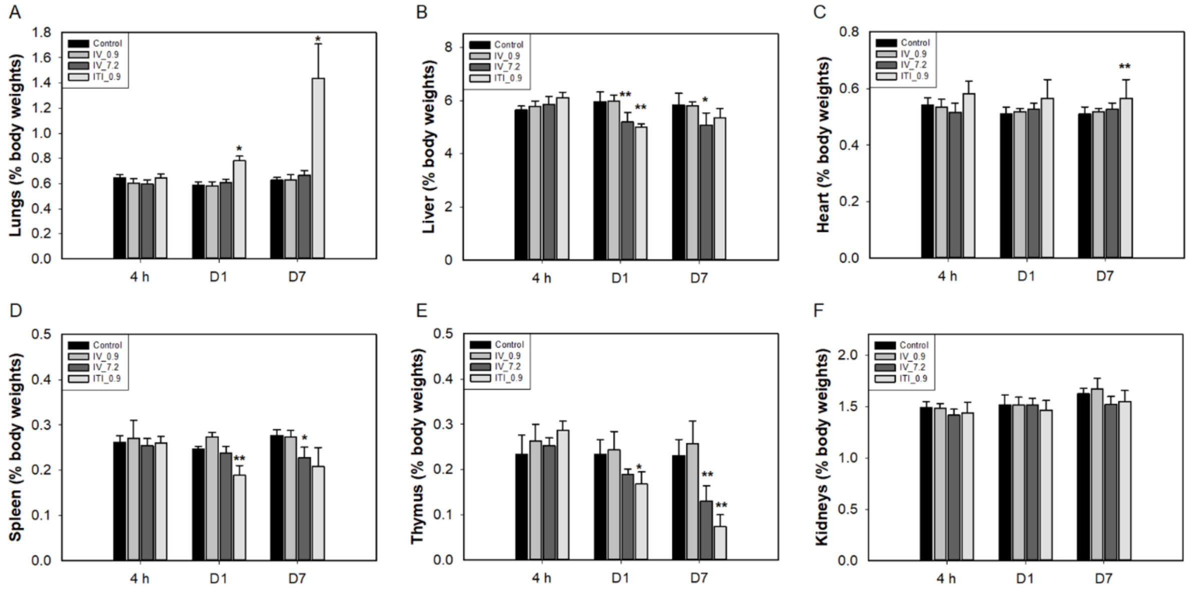

Supplementary Figure S1. Changes in the relative organ weights (organ weight (g) / terminal body weight (g) X 100 (%)) of mice. Mice were intravenously injected with 0.9 mg/kg or 7.2 mg/kg of PHMG-P or intratracheally instilled with 0.9 mg/kg of PHMG-P. Mice were sacrificed at 4h and days 1and 7 after treatment. Bars represent the mean ± standard deviation (n = 9). Values are significantly different from the control group: * *p* <0.05, ** *p* <0.01.

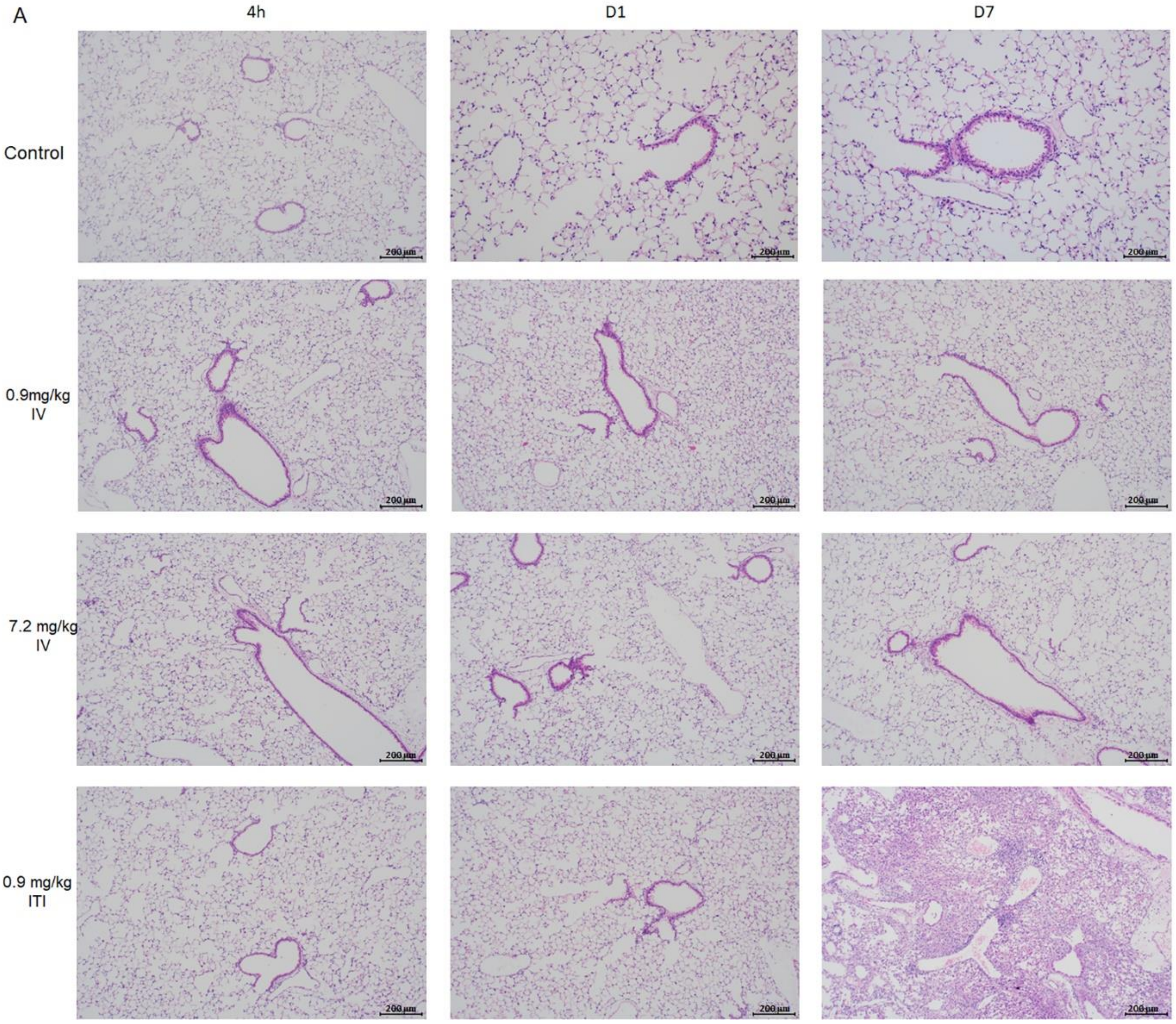

A
4h
D1
D7
Control
0.9mg/kg
IV
7.2 mg/kg
IV
0.9 mg/kg
ITI
200 µm

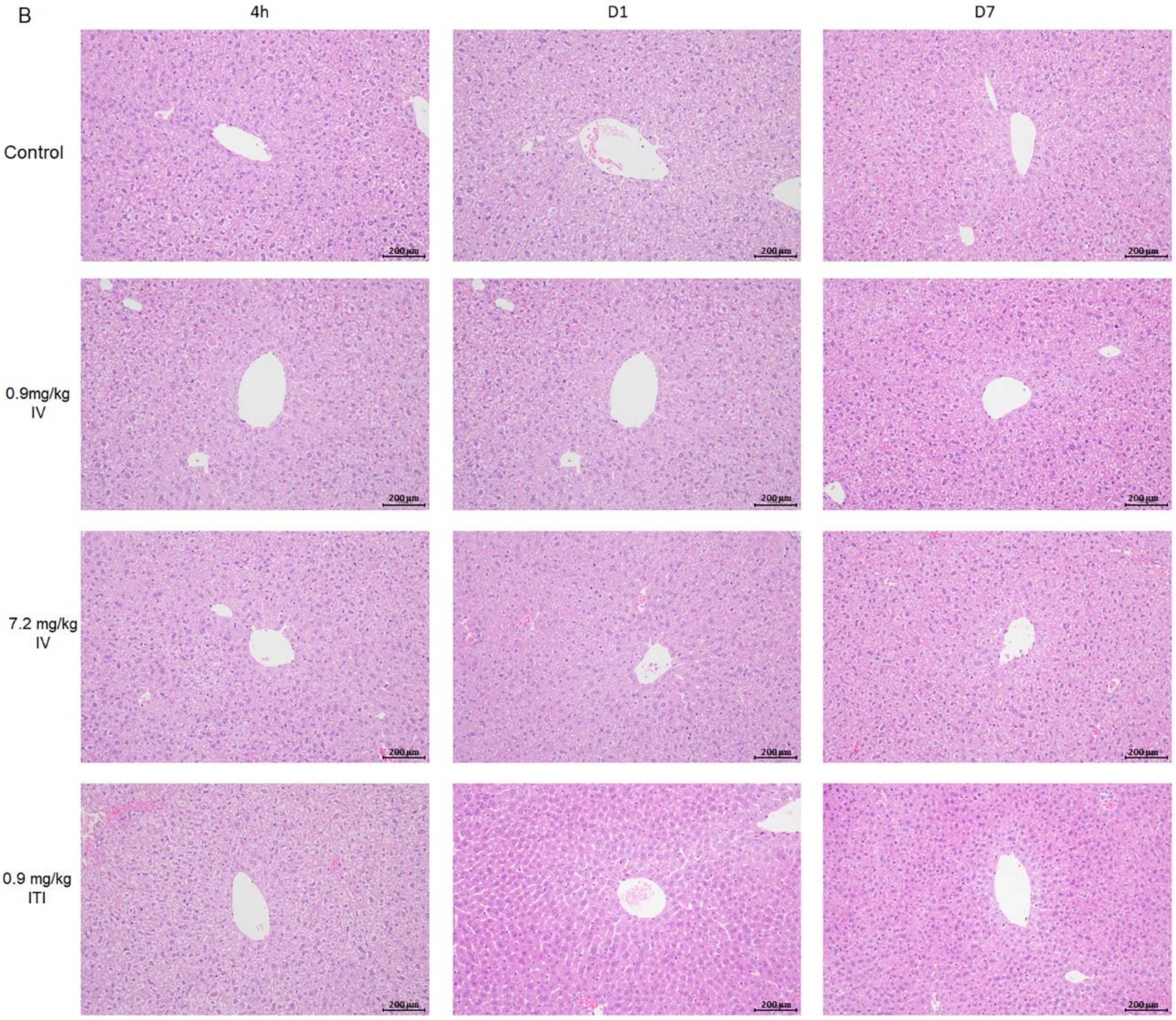
B
4h
D1
D7
Control
0.9mg/kg IV
7.2 mg/kg IV
0.9 mg/kg ITI
200 μm

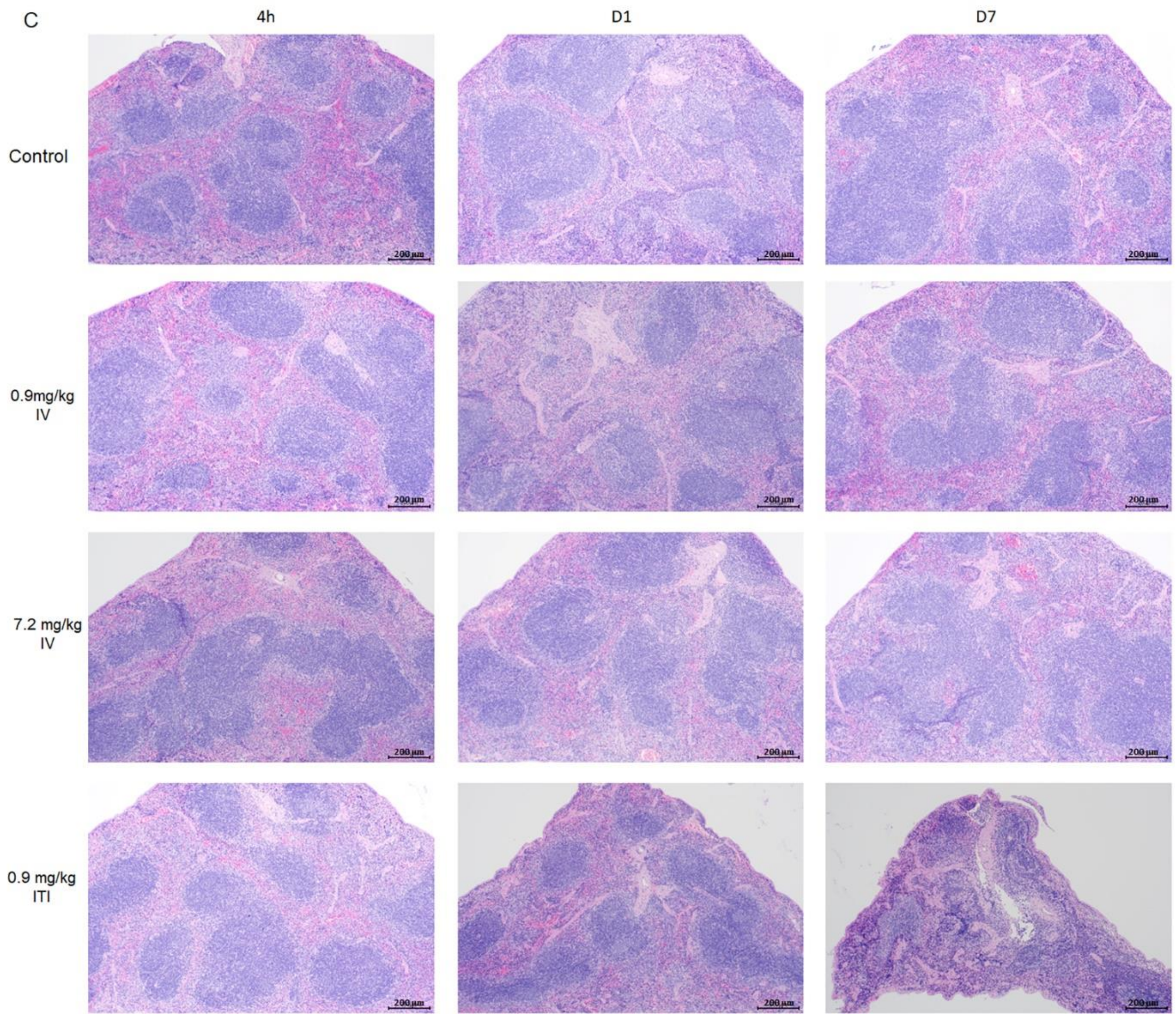

Supplementary Figure S2. Histopathological examination on PHMG-P-treated mice. Representative photographs of lung (A), liver (B), and spleen sections (C) were shown. Scale bar: 200 µm.

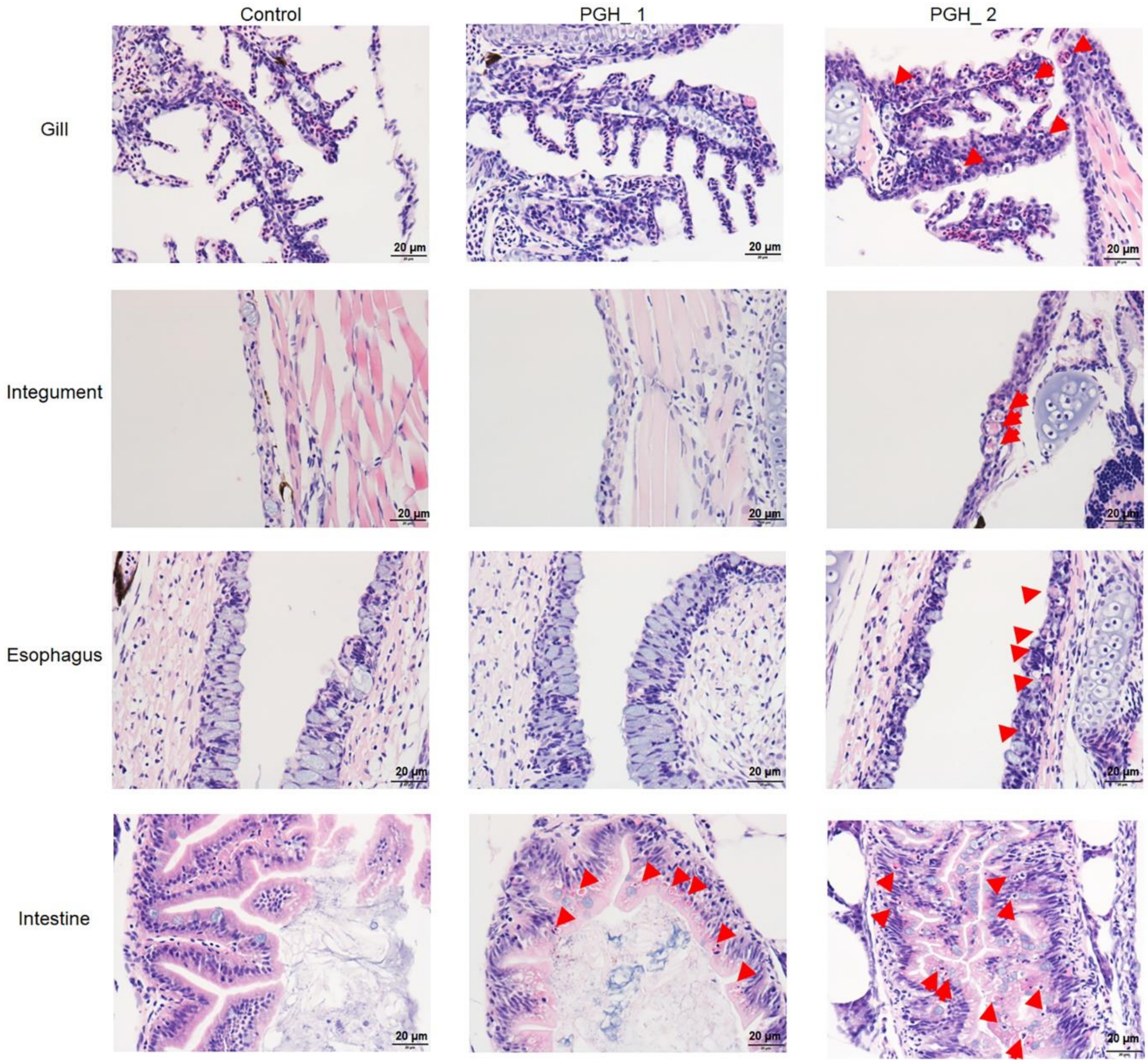

Supplementary Figure S3. Representative photographs of PGH-treated zebrafish. Four-week old zebrafish were exposed to 1, 2, or 2.5 μg /mL PGH for 5 days. All the zebrafish treated with 2.5 μg /mL PGH died on day 1 after exposure. Gills, integument, esophagus, and intestine were shown. Scale bar: 20 μm.

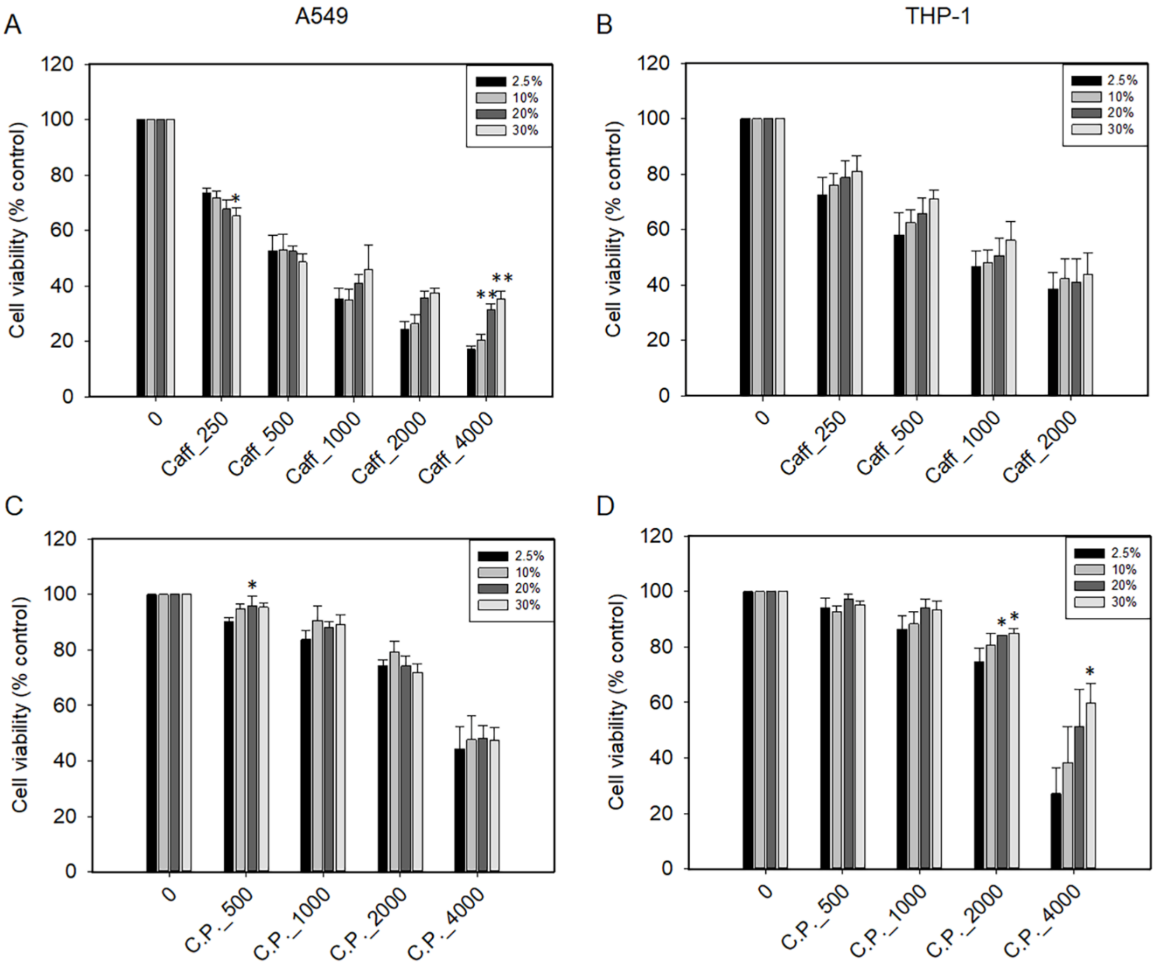

Supplementary Figure S4 Cell viability assay in the cell culture media supplemented with 2.5%, 10%, 20%, or 30% fetal bovine serum (FBS). To evaluate the effect of serum proteins on cell viability, caffeine (Caff) and cyclophosphamide (C.P.), negative control chemicals, were treated in A549 and THP-1 cells. After 24 h, cell viability was measured using CCK-8 reagent. Cell viability was shown as a percentage of control. Data are expressed as mean ± standard deviation of three separate experiments. Values are significant compared to cell viability of 2.5% FBS at each concentration of PHMG-P; * $p<0.05$, ** $p<0.01$
